# *Faecalibacterium prausnitzii* prevents hepatic damage in a mouse model of NASH induced by a high-fructose high-fat diet

**DOI:** 10.3389/fmicb.2023.1123547

**Published:** 2023-03-16

**Authors:** Ji-Hee Shin, Yoonmi Lee, Eun-Ji Song, Dokyung Lee, Seo-Yul Jang, Hye Rim Byeon, Moon-Gi Hong, Sang-Nam Lee, Hyun-Jin Kim, Jae-Gu Seo, Dae Won Jun, Young-Do Nam

**Affiliations:** ^1^Research Group of Personalized Diet, Korea Food Research Institute, Wanju-gun, Jeollabuk-do, Republic of Korea; ^2^R&D Center, Enterobiome Inc., Goyang-si, Republic of Korea; ^3^Division of Applied Life Science (BK21 Four), Institute of Agriculture and Life Science, Gyeongsang National University, Jinju-si, Republic of Korea; ^4^Department of Internal Medicine, Hanyang University, College of Medicine, Seoul, Republic of Korea

**Keywords:** chronic liver disease, non-alcoholic steatohepatitis, gut microbiota, next generation probiotics, *Faecalibacterium prausnitzii*

## Abstract

**Introduction:**

Nonalcoholic steatohepatitis (NASH) is an advanced nonalcoholic fatty liver disease characterized by chronic inflammation and fibrosis. A dysbiosis of the gut microbiota has been associated with the pathophysiology of NASH, and probiotics have proven helpful in its treatment and prevention. Although both traditional and next-generation probiotics have the potential to alleviate various diseases, studies that observe the therapeutic effect of next-generation probiotics on NASH are lacking. Therefore, we investigated whether a next-generation probiotic candidate, *Faecalibacterium prausnitzii*, contributed to the mitigation of NASH.

**Methods:**

In this study, we conducted 16S rRNA sequencing analyses in patients with NASH and healthy controls. To test *F. prausnitzii* could alleviate NASH symptoms, we isolated four *F. prausnitzii* strains (EB-FPDK3, EB-FPDK9, EB-FPDK11, and EB-FPYYK1) from fecal samples collected from four healthy individuals. Mice were maintained on a high-fructose high-fat diet for 16 weeks to induce a NASH model and received oral administration of the bacterial strains. Changes in characteristic NASH phenotypes were assessed via oral glucose tolerance tests, biochemical assays, and histological analyses.

**Results:**

16S rRNA sequencing analyses confirmed that the relative abundance of *F. prausnitzii* reduced significantly in patients with NASH compared to healthy controls (*p* < 0.05). In the NASH mice, *F. prausnitzii* supplementation improved glucose homeostasis, prevented hepatic lipid accumulation, curbed liver damage and fibrosis, restored damaged gut barrier functions, and alleviated hepatic steatosis and liver inflammation. Furthermore, real-time PCR assays documented that the four *F. prausnitzii* strains regulated the expression of genes related to hepatic steatosis in these mice.

**Discussion:**

Our study, therefore, confirms that the administration of *F. prausnitzii* bacteria can alleviate NASH symptoms. We propose that *F. prausnitzii* has the potential to contribute to the next-generation probiotic treatment of NASH.

## 1. Introduction

Nonalcoholic fatty liver disease (NAFLD) refers to a group of diseases, including simple steatosis (in which fat is excessively accumulated in hepatocytes), nonalcoholic steatohepatitis (NASH, with hepatocellular necrosis, inflammation, and fibrosis), and further progressive cirrhosis (Brunt, 2001). Although the pathogenesis of NASH and cirrhosis is not fully understood, the double-hit hypothesis is widely accepted. The first hit comprises fat accumulation in the liver due to insulin resistance, and the second hit consists of lipid peroxidation and inflammatory processes caused by oxidative stress, thereby causing hepatocellular damage and an inflammatory response (Day and James, 1998). However, it is now understood that NAFLD and particularly NASH progression is caused by more complex and diversely parallel metabolic stimuli (known as the multiple parallel hit theory), such as insulin resistance, hormones secreted from the adipose tissue, nutritional factors, gut microbiota, and genetic and epigenetic factors (Arab et al., 2017; Tilg et al., 2021). Moreover, a recent study demonstrated that an imbalance in the intestinal microbiome is associated with liver disease (Goel et al., 2014).

The human intestinal microbiome consists of 100 trillion microorganisms, which is 10 times the number of human somatic and reproductive cells (Backhed et al., 2005; Hooper and Macpherson, 2010). The commensal microbiome metabolizes indigestible compounds, produces vitamins, defends against opportunistic pathogens, and contributes to the development and regulation of mammalian immune systems (Carding et al., 2015; Jandhyala et al., 2015). A balance in the microbial composition is crucial for maintaining the host health. When this balance is disrupted, the host may experience dysbiosis, a decreased resistance to pathogens, the collapse of pathological immune responses, or the onset of severe diseases (Kamada et al., 2013; Carding et al., 2015; Das and Nair, 2019). Several studies have implicated gut microbial dysbiosis in NAFLD and NASH (Zhu et al., 2013; Michail et al., 2015; Boursier et al., 2016). Patients with obesity and NASH exhibited decreased microbial diversity compared to healthy controls (Zhu et al., 2013); the proportion of *Bacteroides* and *Prevotella* species increased significantly in these patients, whereas those of *Blautia* and *Faecalibacterium* decreased (Zhu et al., 2013; Michail et al., 2015; Boursier et al., 2016).

The Food and Agriculture Organization and World Health Organization defined probiotics as “live microorganisms which, when administered in adequate amounts, confer a health benefit on the host” in 2001. Probiotics have the potential to prevent or treat various health problems such as inflammatory bowel disease, obesity, diabetes, and cardiovascular disease by controlling host-gut microbial interactions (Kim et al., 2019). Commercialized *Streptococcus, Lactobacillus*, and *Bifidobacterium* are well-known probiotics that promote an anti-inflammatory environment and assist with gut barrier function (Paolella et al., 2014). Recently, microorganism-based studies have investigated their potential to alleviate chronic liver disease (Li et al., 2003; Ma et al., 2008; Xu et al., 2012; Xin et al., 2014). The administration of VSL#3, a multi-strain formulation that contains a mixture of the aforementioned three bacterial genera, improved the serum alanine aminotransferase (ALT) levels and histological spectrum of liver damage in Lep*^ob/ob^* mice and rats (Li et al., 2003; Ma et al., 2008). In addition, the administration of *Bifidobacterium longum* reduced hepatic fat accumulation irrespective of gut permeability restoration in a rat model (Xu et al., 2012). Further investigations using a high-fat diet mouse model indicated that the administration of *Lactobacillus johnsonii* BS15 also protects against hepatic steatosis and hepatocyte apoptosis (Xin et al., 2014).

Increasing knowledge of the human gut microbiome has changed the paradigm of probiotics and leads to a natural shift to novel therapeutics such as next-generation probiotics (NPGs) and pharmaceuticals using dominant gut microbes such as *Akkermansia muciniphila*, *Faecalibacterium prausnitzii*, and *Prevotella copri* (Chang et al., 2019; Cheng and Xie, 2021; He et al., 2021). Unlike traditional probiotics that are derived from fermented foods, NGPs have been explored in commensal gut microbiota that supports human health (Martín and Langella, 2019). NGPs have been primarily identified through comparisons of microbiota compositions between healthy and unhealthy individuals, and they comprise various genera (Martín and Langella, 2019). Therefore, many NGP candidates have been reported to alleviate various diseases, such as obesity and type 2 diabetes (Munukka et al., 2017; Depommier et al., 2019; López-Moreno et al., 2021). Although these microorganisms are often referred to as novel next-generation therapeutics, studies on the preventative mechanism of these microbes on NASH symptoms are still lacking. In this study, *F. prausnitzii* was selected as a next-generation probiotic candidate in an exploration of the gut microbiota of 45 patients with NASH and 99 healthy controls. We investigated the effect of four *F. prausnitzii* isolates on NASH using high-fat and high-fructose diet mouse models that most closely recapitulate the human phenotype of NASH. NASH symptoms, such as glucose homeostasis, hepatic lipid accumulation, and liver damage, were evaluated. Furthermore, we analyzed gut barrier function and mRNA levels of genes related to liver hepatic steatosis and liver inflammation to explore mechanistic insights into the anti-NASH effect of *F. prausnitzii.*

## 2. Materials and methods

### 2.1. Human participants

Patients diagnosed with NASH based on liver histology were recruited from the Hanyang University College of Medicine (Seoul, Republic of Korea). The diagnostic criteria for NASH were satisfied if patients met the following three conditions: an alcohol consumption of less than 20 g per day, biopsy-proven steatohepatitis, and the absence of other chronic liver diseases. The exclusion criteria comprised any consumption of probiotics or prebiotics within 3 months of the study, pregnancy and/or lactation, or any history of major gastrointestinal surgery. All participants provided written informed consent, and the study was approved by the institutional review board of Hanyang University College of Medicine (IRB number: 2014–03–008-005). Age-and sex-matched healthy subjects recruited in our previous study (Lim M. Y. et al., 2021) were used as the control group in this study.

### 2.2. 16S ribosomal RNA sequencing of stool samples

Fresh stool samples were collected from the participants using OMR-200 fecal sampling kits (OMNIgene GUT; DNA Genotek, Kanata, ON, Canada) and stored at-80°C prior to DNA extraction. Bacterial genomic DNA was extracted from fecal samples according to the instructions of the QIAamp DNA Stool Mini Kit (Qiagen, Hilden, Germany). The V3/V4 hypervariable region of 16S rRNA genes was amplified and sequenced using the Illumina MiSeq 2 × 300 System (Illumina, San Diego, CA, United States) according to the manufacturer’s instructions. Raw sequencing reads were analyzed using the QIIME 2 pipeline (Bolyen et al., 2019). Briefly, raw sequence data were demultiplexed and filtered for quality using the DADA2 plugin (Callahan et al., 2016). Only features belonging to the bacterial domain and in the range of 380–450 base pairs were assessed. *De novo* chimera filtration was performed using the “vsearch uchime-denovo” program (Rognes et al., 2016). Following data filtration, taxonomy was assigned using a pre-trained naive Bayes classifier against Silva-138 reference sequences. Species assignment was conducted with the “vsearch usearch_global” tool and was based on a 99% identity threshold. The dataset was rarefied to the smallest sample before beta diversity analysis. All raw sequencing data presented in this study were deposited in the Sequence Read Archive database under accession number PRJNA901628^1^.

### 2.3. Bacterial strains and culture

*Faecalibacterium prausnitzii* bacteria were isolated from human feces according to the method described by Martín et al. (2017), with some modifications. Human feces were collected from healthy Koreans aged 7–60 years as approved by the Institutional Review Board of Dongguk University Ilsan Hospital in the Republic of Korea (2018–06–001-012). As detailed in a previous study (Lee et al., 2022), we performed polymerase chain reaction (PCR) tests using species-specific primers for *F. prausnitzii* (forward primer: 5’-ACTCAACAAGGAAGTGA-3′; reverse primer: 5’-AATTCCGCCTACCTCTG-3′) to identify the isolates, producing a single band of the available product size (192 bp). Following PCR confirmation, we conducted 16S rRNA gene sequencing using 27F primer (5’-AGAGTTTGATCCTGGCTCAG-3′) and 1492R primer (5’-GGTTACCTTGTTACGACTT-3′). For our bacterial studies, *F. prausnitzii* strains were cultured in soy peptone-based medium containing (per liter): 20 g soy peptone; 10 g yeast extract; 2.5 g K_2_HPO_4_; 0.5 g l-cysteine hydrochloride, and some supplements. The bacteria were cultured at 37°C in an anaerobic chamber containing 90% N_2_, 5% CO_2_, and 5% H_2_. Cells were harvested *via* centrifugation at 12,000 × *g* for 5 min at 4°C. Thereafter, the pellets were resuspended in pre-reduced and sterile phosphate-buffered saline (anaerobic PBS), aliquoted, and stored at-80°C in 20% glycerol.

### 2.4. Antibiotic susceptibility and hemolytic activity

The minimum inhibitory concentrations (MIC) were determined for the isolates of seven antibiotic classes (piperacillin-tazobactam, ceftizoxime, chloramphenicol, clindamycin, meropenem, moxifloxacin, metronidazole, and ciprofloxacin) which are effective against anaerobic bacteria using the Wilkins–Chalgren medium according to Clinical and Laboratory Standards Institute (2017). All MICs were interpreted using the CLSI breakpoints for anaerobes. The hemolytic activity of the isolates was determined using tryptic soy agar containing 5% (v/v) defibrinated sheep blood, with the plates being incubated at 37°C for 24 h under the anaerobic conditions listed in Section 2.3. After incubation, hemolytic activity was evaluated and classified based on red blood cell lysis in the medium surrounding the colonies. Strains with no zones around the colonies (γ-hemolysis) were considered safe.

### 2.5. Study animals and treatments

Six-week-old female C57BL/6 mice were purchased from Daehan Biolink Co., Ltd. (Chungbuk, Korea). The use and care of animals were reviewed and approved by the Institutional Animal Care and Use Committee (IACUC) at Dongguk University (approval number: IACUC-2019-041-1) and conformed with the guidelines of the International Association for the Study of Pain policies on the use of laboratory animals. After 1 week of acclimation, the animals were randomly assigned and housed in standard plastic cages (three mice per cage). A total of 96 mice were randomized into the following eight groups (each group *n* = 12): a normal control (CON), NASH, NASH with silymarin, A2-165, EB-FPDK3, EB-FPDK9, EB-FPDK11, or EB-FPYYK1 group. All groups were maintained for 16 weeks under different regimens. The normal group was fed a low-fat diet (10 kcal% fat; Research Diets, Inc., NJ, United States) and had free access to plain tap water. NASH model groups were fed a high-fat diet (60 kcal% fat; Research Diets, Inc.) and had free access to water enriched with 30% fructose (high-fructose, HF). Mice in the remaining groups started receiving oral administrations at 8 weeks, consisting of silymarin (NASH with silymarin group) or 1 × 10^8^ CFU of *F. prausnitzii* strains (A2-165, EB-FPDK3, EB-FPDK9, EB-FPDK11, and EB-FPYYK1 groups). The weight and calorific intake of all mice were measured weekly. After the experiment, the mice were anesthetized to collect blood samples (see Sections 2.6 and 2.7) and euthanized before removal of the spleen, liver, and large intestine. Fresh spleens and livers were weighed. The livers and large intestine were partially sectioned and fixed for histological analysis (Section 2.8), with the remaining tissues stored at-80°C for RNA analysis (Section 2.9).

### 2.6. Oral glucose tolerance test

An OGTT was performed during the last week of the study. After being subjected to 14 h of fasting, the mice were administered oral glucose (2 g/kg), and blood was obtained from the tail vein 0, 30, 60, and 120 min after glucose treatment. Glucose levels (mg/dL) were measured using Accu-Chek test strips on an Accu-Chek Active blood glucose meter (Roche Diagnostics, Rotkreuz, Switzerland). The glucose area under the curve was calculated by plotting the glucose concentration as a function of time (min).

### 2.7. Biochemical analysis

Whole blood samples were centrifuged (2000 × *g* for 15 min at 4°C) to separate the serum. The serum triglyceride (TG), total cholesterol (TC), aspartate aminotransferase (AST), and ALT levels were measured using assay kits (Asan Pharmaceutical, Seoul, Korea). Lipids were extracted from the liver tissue according to the Folch protocol (Folch et al., 1957).

### 2.8. Histological analysis of liver and large intestine tissues

The liver and large intestine were fixed in neutral-buffered 10% formalin solution, embedded in paraffin wax, and sectioned at a thickness of 4 μm using a microtome. Hematoxylin and eosin (H&E) staining, Sirius Red staining, and α-SMA (ab5694; Abcam, Cambridge, MA, United States) immunohistochemistry (IHC) were performed on the liver sections. To assess the degree of steatosis, lobular inflammation, and hepatocyte ballooning, a NAFLD Activity Score (NAS) was assigned to each group. The NAS system was proposed by the National Institute of Diabetes and Digestive and Kidney Diseases–NASH Clinical Research Network, and the score range is described in Table 1. The thickness of the mucosa and muscularis externa in the large intestine tissue sections was measured with a Nikon Eclipse Ni microscope (Nikon Corporation, Tokyo, Japan). Large intestine sections were stained with anti-zonular-1 antibody (67–7,300; Invitrogen, Waltham, MA, United States) and anti-occludin antibody (71–1,500; Invitrogen) for the analysis of tight junctions. The area of liver fibrosis was quantified using ImageJ software (NIH, Bethesda, MD, United States).

**Table 1:** Nonalcoholic fatty liver disease activity scores (NAS).

NAS components		
Item	Score	Extent
Steatosis	0	<5%
	1	5–33%
	2	>33–66%
	3	>66%
Lobular inflammation	0	No Foci
	1	<2 foci at ×200
	2	2–4 foci at ×200
	3	>4 foci at ×200
Hepatocyte ballooning	0	None
	1	Few balloon cells
	2	Many cells/prominent ballooning

### 2.9. Real-time PCR for assessing mRNA expression

Total RNA was extracted from homogenized liver and large intestine tissues using TRIzol Reagent (Life Technologies, Carlsbad, CA, United States) and purified using RNA PureLink RNA Mini Kits (Thermo Fisher Scientific, Waltham, MA, United States) according to the manufacturer’s instructions. Complementary DNA (cDNA) was synthesized with a reaction micture of volume 20 μl, containing 2 μg of pure RNA, oligo dT primer (M-MLV cDNA Synthesis Kit, Enzynomics), and an M-MLV reverse transcriptase (M-MLV cDNA Synthesis Kit, Enzynomics, Daejeon, Korea) according to the manufacturer’s instructions. For quantitative real-time polymerase chain reaction (qRT–PCR), the cDNA (2 μl) was mixed with primer pairs (250 nM each) and 10 μl of qPCR 2× SYBR Green Premix (Enzynomics, Daejeon, Korea) in reaction mixture of volume 20 μl. After initial denaturation at 95°C for 10 min, cDNA was amplified for 40 cycles of denaturation (95°C, 15 s) and annealing (60°C, 1 min) using QuantStudio3 (Applied Biosystems). The results were normalized to glyceraldehyde-3-phosphatase dehydrogenase (GAPDH). All primer sequences are listed in Table 2.

**Table 2:** Forward primer (F) and reverse primer (R) sequences used for PCR analyzes.

Target	NCBI Gene accession number	Primer sequence (5′ to 3′)
GAPDH	NM_001411843	F: GAC ATC AAG AAG GTG GTG AAG CAG
		R: ATA CCA GGA AAT GAG CTT GAC AAA
PPAR-γ	NM_011146	F: CAA GAA TAC CAA AGT GCG ATC AA
		R: GAG CTG GGT CTT TTC AGA ATA ATA AG
FAS	NM_007987	F: TAT CAA GGA GGC CCA TTT TGC
		R: TGT TTC CAC TTC TAA ACC ATG CT
LPL	NM_008509	F: CTC TGT ATG GCA CAG TGG CT
		R: TCC ACC TCC GTG TAA ATC AA
SREBP-1c	NM_001358315	F: GCT ACC GGT CTT CTA TCA ATG
		R: GCA AGA AGC GGA TGT AGT C
CD36	NM_001159558	F: GCT TGC AAC TGT CAG CAC AT
		R: GCC TTG CTG TAG CCA AGA AC
FATP 5	NM_009512	F: GAC TTT TGA TGG GCA GAA GC
		R: GGG CCT TGT TGT CCA GTA TG
L-FABP	NM_017399	F: ACC TCA TCC AGA AAG GGA AGG
		R: ACA ATG TCG CCC AAT GTC ATG
TNF-α	NM_001278601	F: CCG ATG GGT TGT ACC TTG TC
		R: GGGCTGGGTAGAGAATGGAT
TLR4	NM_021297	F: CCT CTG CCT TCA CTA CAG AGA CTT T
		R: TGT GGA AGC CTT CCT GGA TG
MCP1	NM_011333	F: TTA AAA ACC TGG ATC GGA ACC A
		R: GCA TTA GCT TCA GAT TTA CGG G
IL-6	NM_031168	F: TCC TAC CCC AAT TTC CAA TGC
		R: CAT AAC GCA CTA GGT TTG CCG
Col1a1	NM_007742	F: GCT CCT CTT AGG GGC CAC T
		R: CCA CGT CTC ACC ATT GGG G
TIMP-1	NM_011593	F: CGA GAC CAC CTT ATA CCA GCG
		R: GGC GTA CCG GAT ATC TGC G
ZO-1	NM_00163574	F: GCC GCT AAG AGC ACA GCA A
		R: TCC CCA CTC TGA AAA TGA GGA
Occludin	NM_001360538	F: ATG TCC GGC CGA TGC TCT C
		R: TTT GGC TGC TCT TGG GTC TGT AT
GPR43	NM_001168512	F: GGC TTC TAC AGC AGC ATC TA
		R: AAG CAC ACC AGG AAA TTA AG
GLP-1	NM_008100	F: GGC ACA TTC ACC AGC GAC TAC
		R: CAA TGG CGA CTT CTT CTG GG

### 2.10. Analysis of gut microbial populations from the cecum

Using the QIAamp® DNA Stool Mini Kit, DNA was extracted from cecal samples of control, NASH-induced, EB-FPDK9, and EB-FPDK11-treated mice (*n* = 7 per group). 16S rRNA hypervariable regions in V1–V2 were amplified by using primers containing unique 10-base barcodes and sequenced by using the Ion Torrent PGM system according to manufacturer’s instructions. Quality-filtered raw sequence reads were clustered into amplicon sequence varients (ASVs) using the SILVA rRNA gene database with a 99% sequence identity threshold. The software Quantitative Insights into Microbial Ecology 2 (QIIME2) was used to select representative reads and calculate alpha-and beta-diversity. The linear discriminant analysis (LDA) effect size (LEfSe) was used to identify taxa with varying abundances between groups, with a logarithmic LDA score threshold of 2.0 and an alpha value of 0.05 for the factorial Kruskal-Wallis test.

### 2.11. Analysis of short chain fatty acids from the cecum

To extract short chain fatty acids (SCFAs), the cecum (50 mg) was mixed with 800 μl of distilled water and 10 μl of 5 M HCl and after adding 400 μl of ether, the mixture was shaken at 4°C for 5 min. After spin down, 200 μl of ether layer was derivatized by adding 20 μl of N, O-bis (trimethylsilyl) trifluoroacetamide (BSTFA) at 70°C for 20 min and then incubated at 37°C for 2 h. The derivatized SCFAs were analyzed using a GC/MS system (Shimadzu Corp., Kyoto, Japan) equipped with a DB-5MS column (30 m × 0.25 mm, 0.25 μm film thickness, Agilent Technologies, Santa Clara, CA, United States) at a split ratio of 1: 50. The injector temperature was set at 200°C, and helium was used as the carrier gas at a flow rate of 0.89 ml/min. The oven temperature program was set as holding at 40°C for 2 min, increasing from 40 to 70°C at a rate of 10°C/min, increasing from 70 to 85°C at a rate of 4°C/min, increasing from 85 to 110°C at a rate of 6°C/min, increasing from 110 to 290°C at a rate of 90°C/min, and holding 290°C for 5 min. The effluent was detected using a GCMS-TQ 8030 MS (Shimadzu Corp.) system with selected ion monitoring (SIM). The ion source and interface temperatures were 200 and 250°C, respectively, and detector voltage was 0.1 kV. SCFAs were detected by SIM mode with m/z 117, 131, and 145 of acetic acid, propionic acid, and butyric acid, respectively. Authentic standard SCFAs were used to quantitative analysis of SCFAs.

### 2.12. Identification of the microbial anti-inflammatory molecule protein

MAM sequences were identified from four *F. prausnitzii* genomes (EB-FPDK3, EB-FPDK9, EB-FPDK11, and EB-FPYYK1) by BLAST search with low e-values against the MAM sequence from A2-165 as the query (Quévrain et al., 2016; Auger et al., 2022; Hong and Nam, n.d.). Translated MAM sequences were aligned by using Clustal Omega (Sievers et al., 2011) to investigate MAM-derived peptides which were previously identified as pep1–5 (Quévrain et al., 2016). Further, homology models of MAM proteins derived from four *F. prausnitzii* strains were provisionally built using the Modeler program (Webb and Sali, 2016).

### 2.13. Statistical analyzes

All data are expressed as the arithmetic mean ± standard error of the mean (SEM). We used GraphPad Prism 7 (GraphPad, San Diego, CA, United States) for all statistical analyzes, comprised of Mann–Whitney *U* tests (two-tailed) or Kruskal–Wallis one-way ANOVAs, with Dunn’s test to correct for multiple comparisons (unless indicated otherwise). We considered a *p* value <0.05 to indicate statistical significance.

## 3. Results

### 3.1. Gut microbiota composition in patients with NASH and healthy individuals

**Figure 1:**
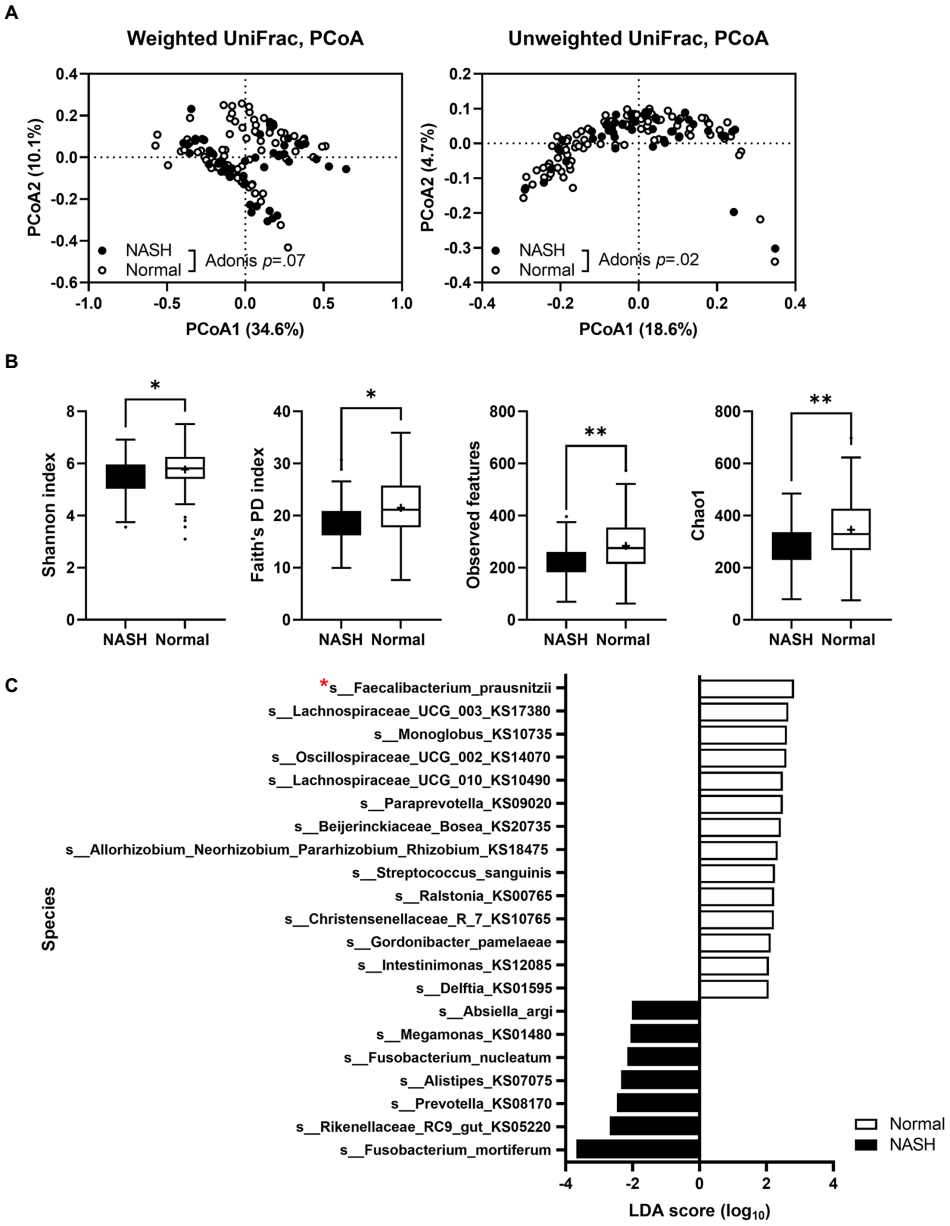
A comparison of gut microbiota in patients with NASH (*n* = 45) and sex- and age-matched healthy individuals (*n* = 99). **(A)** Principal component analysis plot comparing the microbiota of patients with NASH and healthy individuals using unweighted and weighted UniFrac distances. Adonis tests confirmed significant differences between the two groups. **(B)** A comparison of the alpha diversity index between fecal samples of patients with NASH and healthy individuals (Mann–Whitney *U* test; *p ≤ 0.05, ∗∗*p* < 0.01). **(C)** A species-level comparison of the relative abundance of gut microbiota between participants with NASH and healthy individuals. NASH: nonalcoholic steatohepatitis.

To explore the dysbiosis of gut microbiota in NASH, we performed 16S rRNA gene amplicon sequencing on fecal samples from patients with NASH and compared the results with those obtained for healthy individuals. We used our previously published data (Lim M. Y. et al., 2021) on the gut microbiota composition of healthy Korean subjects (*n* = 99), who were age-and sex-matched with patients with NASH (*n* = 45). Gut microbiota compositional discrimination was observed *via* unweighted UniFrac principal component analysis between the NASH and healthy control groups (Figure 1A). Bacterial diversity (according to the Shannon index, Faith’s PD index, observed features, and Chao1 index) in NASH patients was significantly lower compared to the healthy controls (Figure 1B). We also observed dysbiosis in the gut microbiota profile, particularly deficient *F. prausnitzii* and abundant *Fusobacterium mortiferum* (Figure 1C). As a next step, we found that two ASVs belonging to *F. prausnitzii*, 5fdd92ad3225b67f02453b5c4590b968 and 692ed0000e9f6e5d47f92a7c59d88434, showed significant differences between the groups (Supplementary Figure S1A). Using these ASVs, strains EB-FPDK3 and EB-FPDK9 were matched to ASV 692ed0000e9f6e5d47f92a7c59d88434, whereas strains EB-FPDK11 and EB-FPYYK1 were matched to ASV 5fdd92ad3225b67f02453b5c685 with only minor variations (Supplementary Figure S1B). Therefore, we hypothesized that restoration of these strains in the gut could alleviate NASH symptoms. To test this hypothesis, we isolated four *F. prausnitzii* strains (EB-FPDK3, EB-FPDK9, EB-FPDK11, and EB-FPYYK1) from fecal samples collected from four healthy individuals (Supplementary Figure S2). Before administering these strains to mice, we confirmed their basic safety *via* laboratory tests for hemolytic activity and antibiotic susceptibility. The hemolytic activities of five strains (the four isolates plus the type strain, A2-165) were evaluated on blood agar plates. None of the tested strains showed α-hemolytic or β-hemolytic activities when grown on blood agar plates. All five strains showed γ-hemolytic activity, that is, negative or no hemolytic activity. Susceptibility to antimicrobial agents was assessed according to the CLSI guidelines. All strains, including the type strain A2-165, were susceptible to clindamycin (MICs <0.125) and metronidazole (MICs ranging from ≤0.125 to 4 μg/ml). However, all strains were resistant to meropenem (MICs >64 μg/ml) and fluoroquinolones (moxifloxacin and ciprofloxacin, MICs ranging from 8 to >32 μg/ml; Supplementary Table S1).

### 3.2. *Faecalibacterium prausnitzii* supplementation improves glucose homeostasis in NASH mice

A high-fructose and high-fat (HFHF) diet is widely used to induce NASH in animal models to achieve a state that most closely resembles the human NAFLD (Rafiq et al., 2009; Im et al., 2021). To examine the effect of *F. prausnitzii* on NASH, five *F. prausnitzii* strains (comprising the reference strain A2-165 and four *F. prausnitzii* isolates) were orally administered to HFHF-fed mice for 9 weeks. Silymarin was used as a positive control; it contains a mixture of flavonolignans extracted from milk thistle and has been used as a natural drug against liver disease for centuries (Kim et al., 2012; Ni and Wang, 2016).

**Figure 2:**
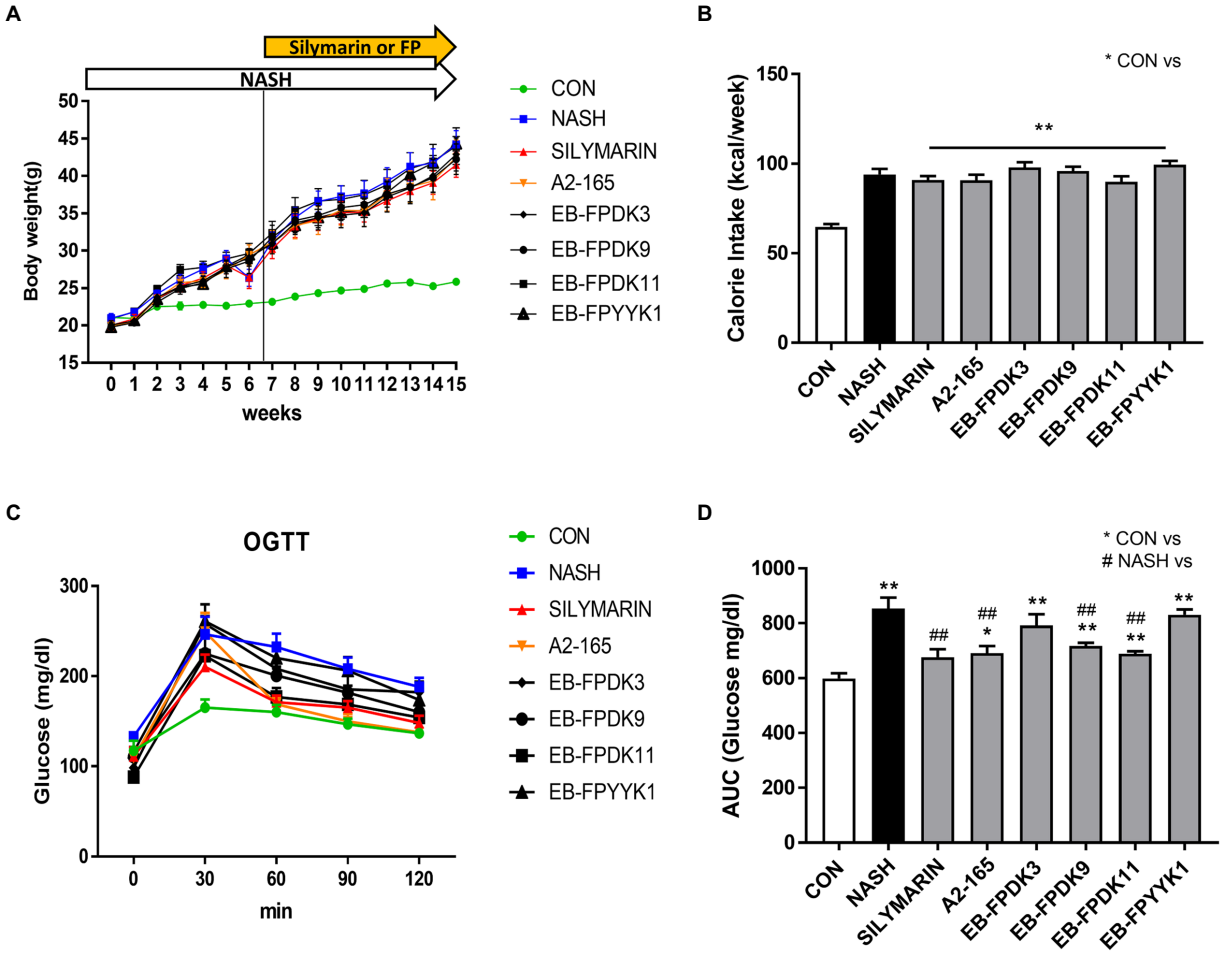
*Faecalibacterium prausnitzii* improves glucose levels in mouse models of NASH induced with a high-fructose and high-fat diet. **(A)** Body weight changes across 12 weeks. **(B)** Weekly calorie intake. **(C)** Oral glucose tolerance test results. **(D)** Glucose area under the curve with glucose concentration as a function of time. The data are presented as mean ± SEM (*n* = 12). Statistical analyzes were performed using Mann–Whitney *U* test (two tailed) **(B,D)**. ∗∗*p* < 0.01 versus the CON group; ## *p* < 0.01 versus the NASH group. CON: control, NASH: nonalcoholic steatohepatitis.

HFHF feeding of mice in the NASH group led to a significant increase in body weight and calorie intake compared to mice following a normal diet in the CON group (Figures 2A,B). There were no significant differences in body weight or calorie intake in any of the treatment groups (referring to the groups in which either silymarin or a bacterial strain was administered) compared to the NASH group. To confirm the effect of *F. prausnitzii* on glucose homeostasis, an OGTT was performed after 16 weeks using blood from mice that had been subjected to a fasting state for 14 h. The blood glucose level reached its highest level 30 min after glucose administration and gradually decreased (Figure 2C). The area under the receiver operating characteristic curve was significantly higher in the NASH group than in the CON group (Figure 2D). Silymarin, A2-165, EB-FPDK9, and EB-FPDK11 supplementation significantly reduced the blood glucose levels compared to those in the NASH group (*p* < 0.05). This indicates that A2-165, EB-FPDK9, and EB-FPDK11 bacterial strains were effective at improving glucose tolerance.

### 3.3. *Faecalibacterium prausnitzii* prevents hepatic lipid accumulation in NASH mice

**Figure 3:**
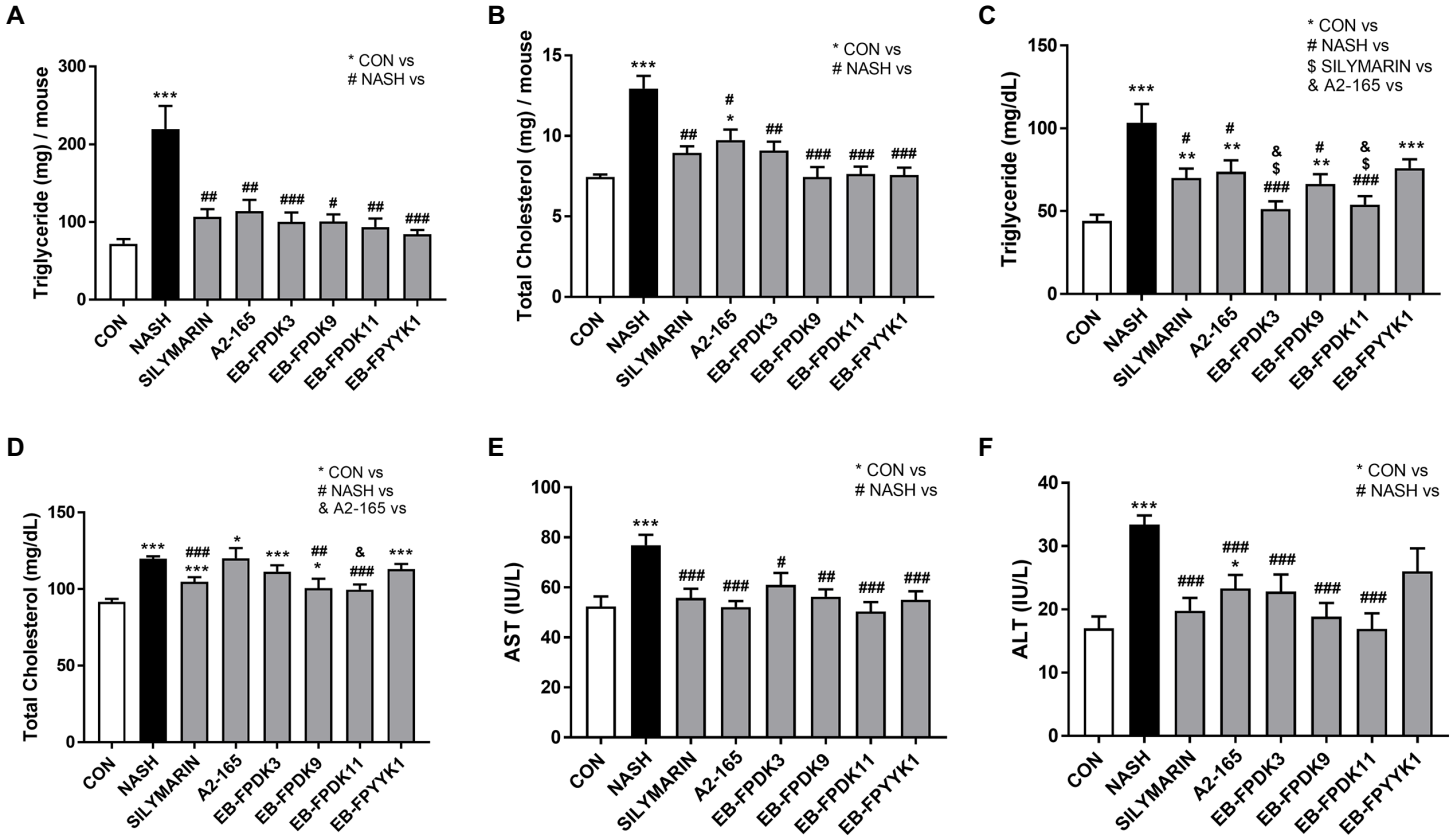
Effects of *Faecalibacterium prausnitzii* in preventing lipid accumulation in and damage to the liver. **(A)** TG and **(B)** TC concentrations in the liver. **(C)** TG and **(D)** TC concentrations in serum. **(E)** Serum AST and **(F)** serum ALT levels. The data are presented as the mean ± SEM (*n* = 12). Statistical analyzes were performed using Kruskal–Wallis one-way ANOVA with Dunn’s test **(A,B)** and the Mann–Whitney *U* test (two-tailed) **(C–F)**. ∗*p* < 0.05, ∗∗*p* < 0.01, ∗∗∗*p* < 0.001 versus the CON group; #*p* < 0.05, ##*p* < 0.01, ###*p* < 0.001 versus the NASH group; $*p* < 0.05 versus the silymarin group; &*p* < 0.05 versus the A2-165 group. TG, triglycerides; TC, total cholesterol; AST, aspartate aminotransferase; ALT, alanine aminotransferase.

NASH mouse models are characterized by the accumulation of hepatic lipids (Saito et al., 2015). To investigate lipid accumulation in the livers of our study animals, we measured the concentrations of TG and TC. Their levels in both the liver and serum were significantly increased in the NASH group compared to the respective levels in the CON group (Figures 3A–D). All treatments significantly decreased TG and TC levels in the liver compared to those in the NASH group (Figures 3A,B). Serum TG levels were significantly decreased in the groups administered with silymarin and all *F. prausnitzii* strains except EB-FPYYK1; the largest decreases were recorded in the EB-FPDK3 and EB-FPDK11 groups. Serum TC levels were significantly decreased by silymarin, EB-FPDK9, and EB-FPDK11 treatment (Figures 3C,D). Serum AST and ALT levels were significantly higher in the NASH group than in the CON group (Figures 3E,F). Compared to those in the NASH group, the serum AST levels were significantly lower in all experimental groups, while the serum ALT levels were significantly decreased in all experimental groups except EB-FPYYK1.

### 3.4. *Faecalibacterium prausnitzii* prevents liver damage and fibrosis in NASH mice

**Figure 4:**
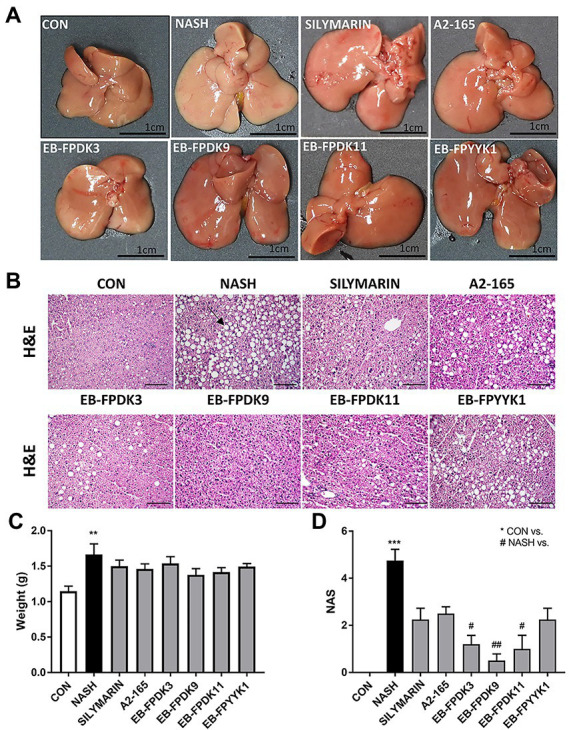
Effects of *Faecalibacterium prausnitzii* in preventing lipid accumulation in and damage to the liver. **(A)** Photographs of mice liver. **(B)** Microscopic images of liver tissue after H&E staining. Scale bar: 0.1 mm. **(C)** Weights of mice liver samples. **(D)** NAFLD Activity Score (NAS). The data are presented as the mean ± SEM (*n* = 12). The black arrow indicates the lipid deposition site in the liver tissue Statistical analyzes were performed using Kruskal–Wallis one-way ANOVA with Dunn’s test, ∗∗*p* < 0.01, ∗∗∗*p* < 0.001 versus the CON group; #*p* < 0.05, ##*p* < 0.01 versus the NASH group. NAFLD: nonalcoholic fatty liver disease, NASH: nonalcoholic steatohepatitis.

Liver images were captured to investigate the extent of liver damage. The livers of mice in the NASH group displayed an enlarged volume of lipid accumulation characterized by a yellow color and hard texture compared to the CON group, in which hepatic lipids exhibited a soft texture with a smooth, red-brown surface (Figures 4A,C). For histopathological analysis, microscope images were obtained of H&E-stained liver tissues (Figure 4B). In the NASH group, we observed microvascular steatosis by lipid deposition and abnormal hepatocyte morphology, such as lobular inflammation and ballooning. When each group was scored according to the NAS criteria listed in Table 1, no abnormal morphology was observed in the CON group, whereas the NAS scores of the EB-FPDK3, EB-FPDK9, and EB-FPDK11 groups were significantly lower than that of the NASH group (Figure 4D). These results show that the oral administration of *F. prausnitzii* strains reduces the amount of lipid accumulation in and subsequent damage to the liver, as induced by NASH.

**Figure 5:**
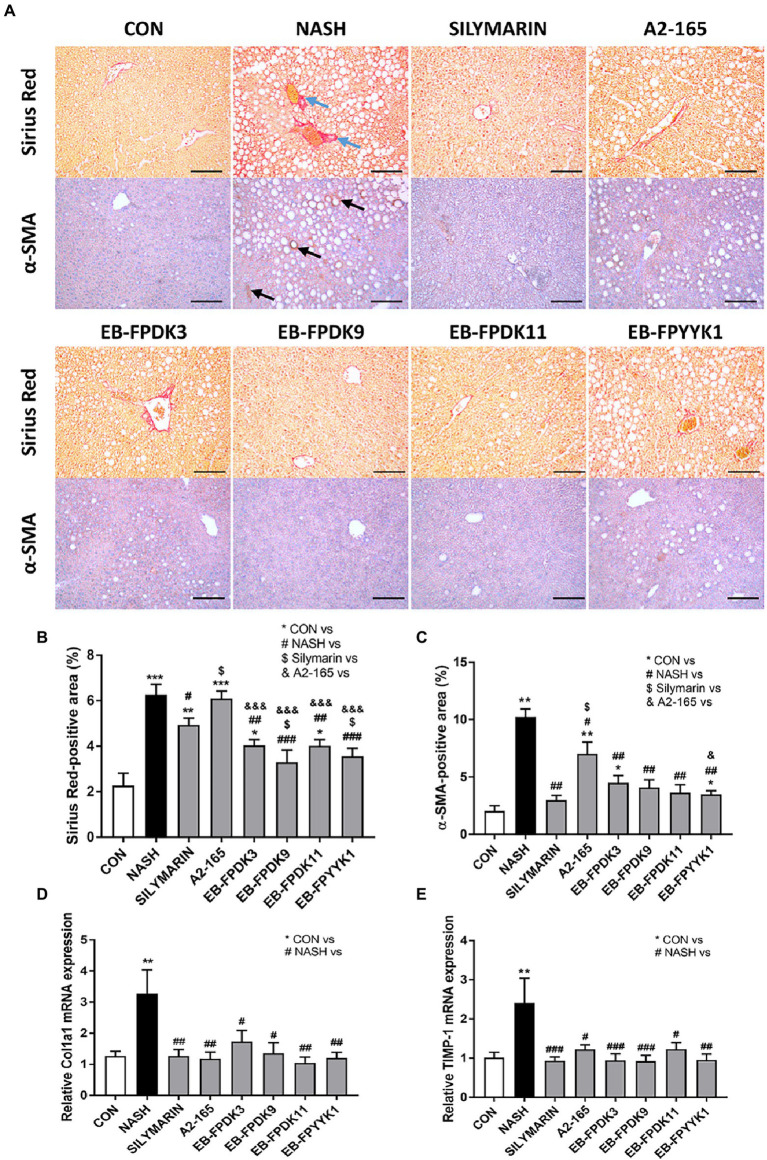
*Faecalibacterium prausnitzii* prevents the development of fibrosis in the liver. **(A)** Microscope images of Sirius Red- and α-SMA-stained liver tissues for immunohistochemistry assays. Scale bar: 0.1 mm. **(B,C)** Areas stained positive for Sirius Red and α-SMA in the liver. mRNA levels of hepatic fibrosis markers **(D)** Col1a1 and **(E)** TIMP-1. The data are presented as the mean ± SEM (*n* = 12). Statistical analyzes were performed using the Mann–Whitney *U* test (two-tailed) ∗*p* < 0.05, ∗∗*p* < 0.01, ∗∗∗*p* < 0.001 versus the CON group; #*p* < 0.05, ##*p* < 0.01, ###*p* < 0.001 versus the NASH group; $*p* < 0.05 versus the silymarin group, &*p* < 0.05, &&&*p* < 0.001 versus the A2-165 group. α-SMA, alpha-smooth muscle actin; Col1a1, collagen type 1 a 1; TIMP-1, metallopeptidase inhibitor 1; CON, control; NASH, nonalcoholic steatohepatitis.

The progression to fibrosis and cirrhosis is a key challenge in human NAFLD. To assess the degree of fibrosis that NASH can induce in the liver, we performed Sirius Red assays (Figures 5A–C). By measuring the Sirius Red-positive areas of livers with ImageJ software, hepatic fibrosis progression was mainly observed in the periportal region of all mouse groups. The livers of NASH group mice showed a significantly larger Sirius red-positive area than those of the EB-FPDK3, EB-FPDK9, EB-FPDK11, EB-FPYYK1, and silymarin groups (Figures 5A,B). Our IHC assay used α-SMA as a marker for evaluating stellate cell activation and fibrosis progression (Akpolat et al., 2005). It identified α-SMA-positive areas predominantly in lipid-accumulated foci. These regions were significantly larger in the NASH group than in the CON group, while they were significantly smaller in all treatment groups compared to the NASH group (Figures 5A,C). We also found that the expression levels of collagen type 1 a 1 and metallopeptidase inhibitor 1 were significantly decreased by *F. prausnitzii* infection (Figures 5D,E). These results indicate a reduction in fibrosis and collagen deposition in the liver after the oral administration of *F. prausnitzii*.

### 3.5. *Faecalibacterium prausnitzii* improves the damaged gut barrier functions of NASH mice

**Figure 6:**
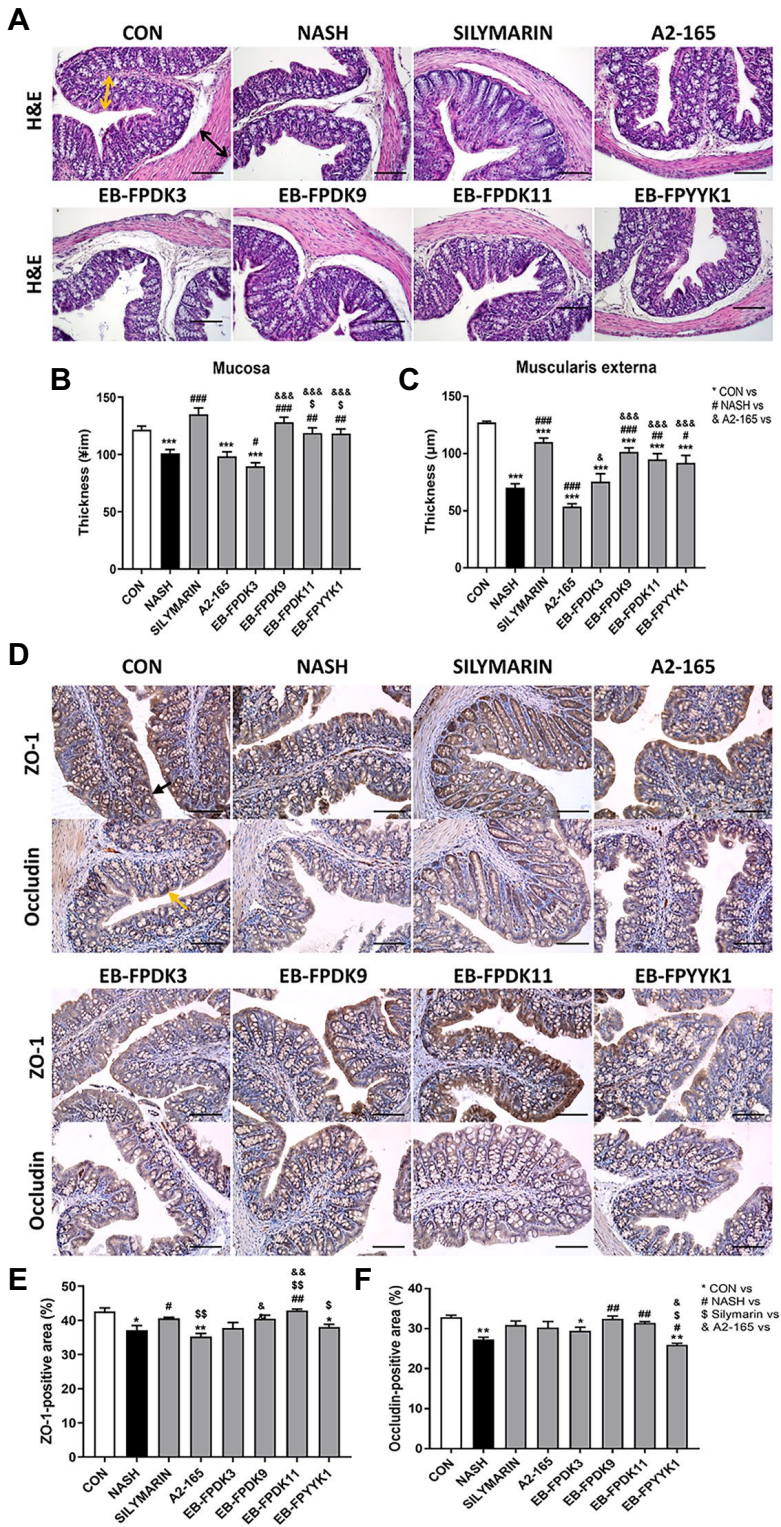
*Faecalibacterium prausnitzii* improves impaired gut barrier functions by modulating the tight junctions in the large intestine. **(A)** Microscope images of large intestine after H&E staining. Scale bar: 0.1 mm. Yellow arrows indicate the thickness of mucosa tissue, and black arrows indicate the thickness of muscularis externa tissue. **(B)** The thickness of the mucosa and **(C)** muscularis externa of the large intestine. **(D)** Microscope images of large intestine tissue after ZO-1 and occludin immunohistochemical staining. Scale bar: 0.1 mm. **(E)** The ZO-1-positive and **(F)** occludin-positive regions. The data are presented as the mean ± SEM (*n* = 12). Statistical analyzes were performed using the Mann–Whitney *U* test (two-tailed) ∗*p* < 0.05, ∗∗*p* < 0.01, ∗∗∗*p* < 0.001 versus the CON group; #*p* < 0.05, ##*p* < 0.01, ###*p* < 0.001 versus the NASH group; $*p* < 0.05, $$*p* < 0.01 versus the silymarin group; &*p* < 0.05, &&*p* < 0.01, &&&*p* < 0.001 versus the A2-165 group. ZO-1, zonula occludens-1; CON, control.

The consumption of an HFHF diet can shift the intestinal luminal composition and increase the risk of gut leakiness (Moreira et al., 2012). Using microscope photographs of H&E-stained large intestine tissues, we measured the thickness of the mucosa and muscularis externa where the damaged colonic barrier appeared thinner (Figures 6A–C). In the NASH group, the mucosa and muscularis externa were significantly thinner compared to those in the CON group. The silymarin group showed significantly increased mucosal and muscularis externa thicknesses compared to the NASH group. Among the *F. prausnitzii* strains, EB-FPDK9, EB-FPDK11, and EB-FPYYK1 showed significant efficacy in improving both the mucosa and muscularis externa thickness.

We investigated the efficacy of barrier function in the large intestine *via* IHC assay, using the expression of zonula occludens-1 (ZO-1) and occludin (OCLN) proteins as indicators (Figures 6D–F). The tight junction protein ZO-1 is essential for barrier function and plays a critical role in the effective mucosal repair (Kuo et al., 2021). In the NASH group, the ZO-1-positive area was significantly smaller compared with that in the CON group, suggesting barrier damage (Figure 6E). Among the *F. prausnitzii* treatments, only that of the EB-FPDK11 strain resulted in a significant increase in ZO-1-positive area compared to the NASH group. OCLN is another tight junction protein that is crucial for maintaining the epithelial barrier (Chelakkot et al., 2018). The OCLN-positive area was significantly smaller in the NASH group than in the CON group, but the area was recovered in mice receiving EB-FPDK9 and EB-FPDK11 treatments (Figure 6F).

### 3.6. *Faecalibacterium prausnitzii* alleviates hepatic steatosis in NASH mice

**Figure 7:**
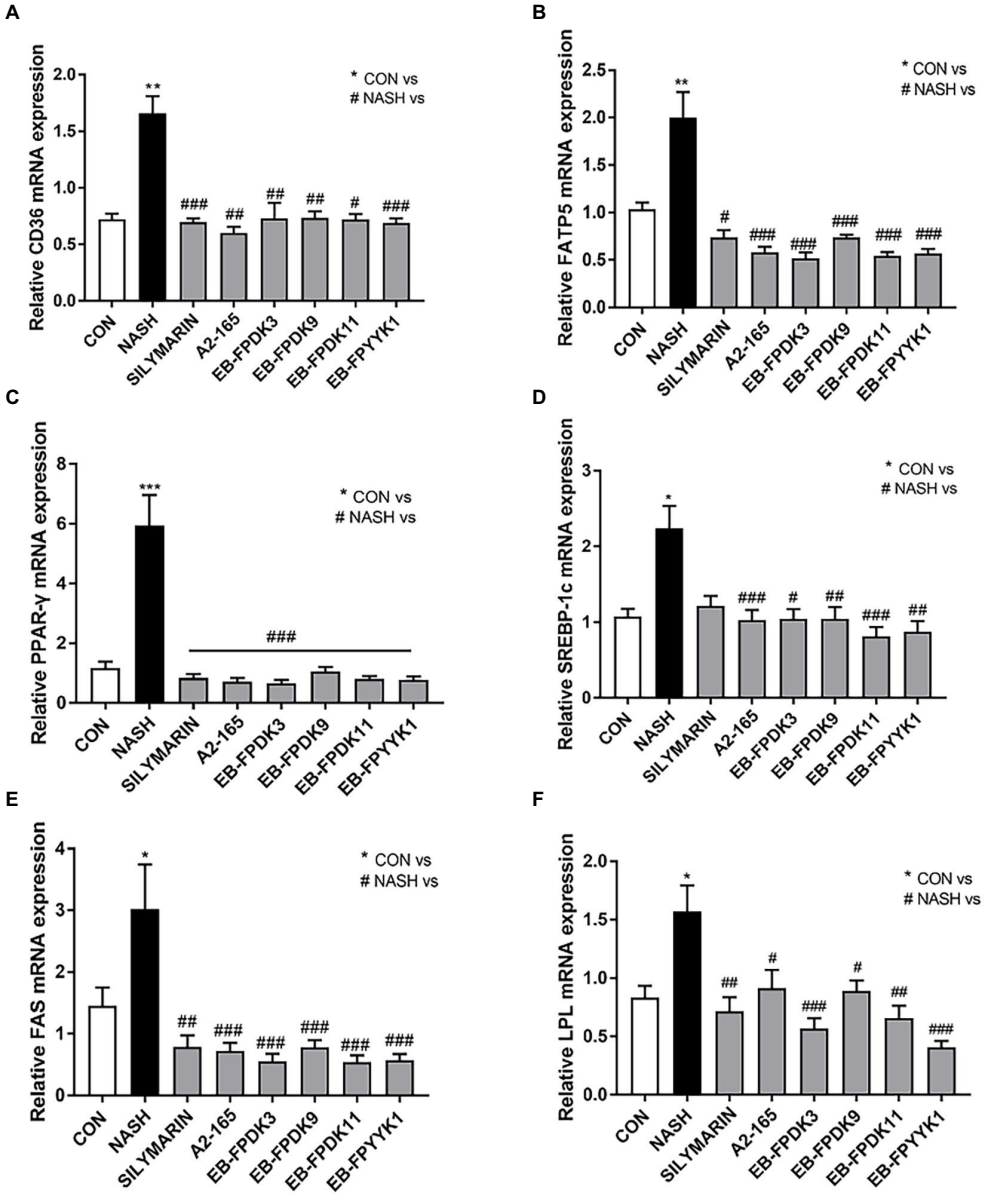
*Faecalibacterium prausnitzii* reduces hepatic steatosis and alleviates liver fat disease. mRNA levels of hepatic steatosis markers **(A)** CD36, **(B)** FATP5, **(C)** PPAR-𝛾, **(D)** SREBP-1c, **(E)** FAS, and **(F)** LPL. Statistical analyzes were performed using Kruskal–Wallis one-way ANOVA with Dunn’s test **(A,D,E)** or Mann–Whitney *U* test (two tailed) **(B,C,F)**. ∗*p* < 0.05, ∗∗*p* < 0.01, ∗∗∗*p* < 0.001 versus the CON group; #*p* < 0.05, ##*p* < 0.01, ###*p* < 0.001 versus the NASH group. FATP5, fatty acid transport protein-5; PPAR-𝛾, peroxisome proliferator-activated receptor gamma; SREBP-1c, sterol regulatory element-binding protein-1c; FAS, fatty acid synthase; LPL, lipoprotein lipase; CON, control; NASH, nonalcoholic steatohepatitis.

To understand the mechanisms underlying the observed effects of *F. prausnitzii* treatment in a NASH mouse model, we evaluated the key signaling pathways involved in the modulation of lipid metabolism, as quantified by real-time reverse-transcriptase PCR. The levels of mRNA expressing the transport proteins CD36 and fatty acid transport protein 5 (FATP5) were approximately 1.6- and 1.99-fold higher in the NASH group than in the CON group, respectively (Figures 7A,B). Additionally, the levels of mRNA expressing peroxisome proliferator-activated receptor gamma (PPAR-γ), sterol regulatory element-binding protein-1c (SREBP-1c), fatty acid synthase (FAS), and lipoprotein lipase (LPL) were also significantly higher in the NASH group. Our PCR results revealed that the expression level of genes involved in lipid metabolism was significantly reduced by oral administration of *F. prausnitzii* (Figures 7C–F). This indicates that *F. prausnitzii* bacteria can regulate the genetic mechanisms underlying lipid metabolism as related to the alleviation of hepatic steatosis in NASH mice.

### 3.7. *Faecalibacterium prausnitzii* alleviates liver inflammation in NASH mice

**Figure 8:**
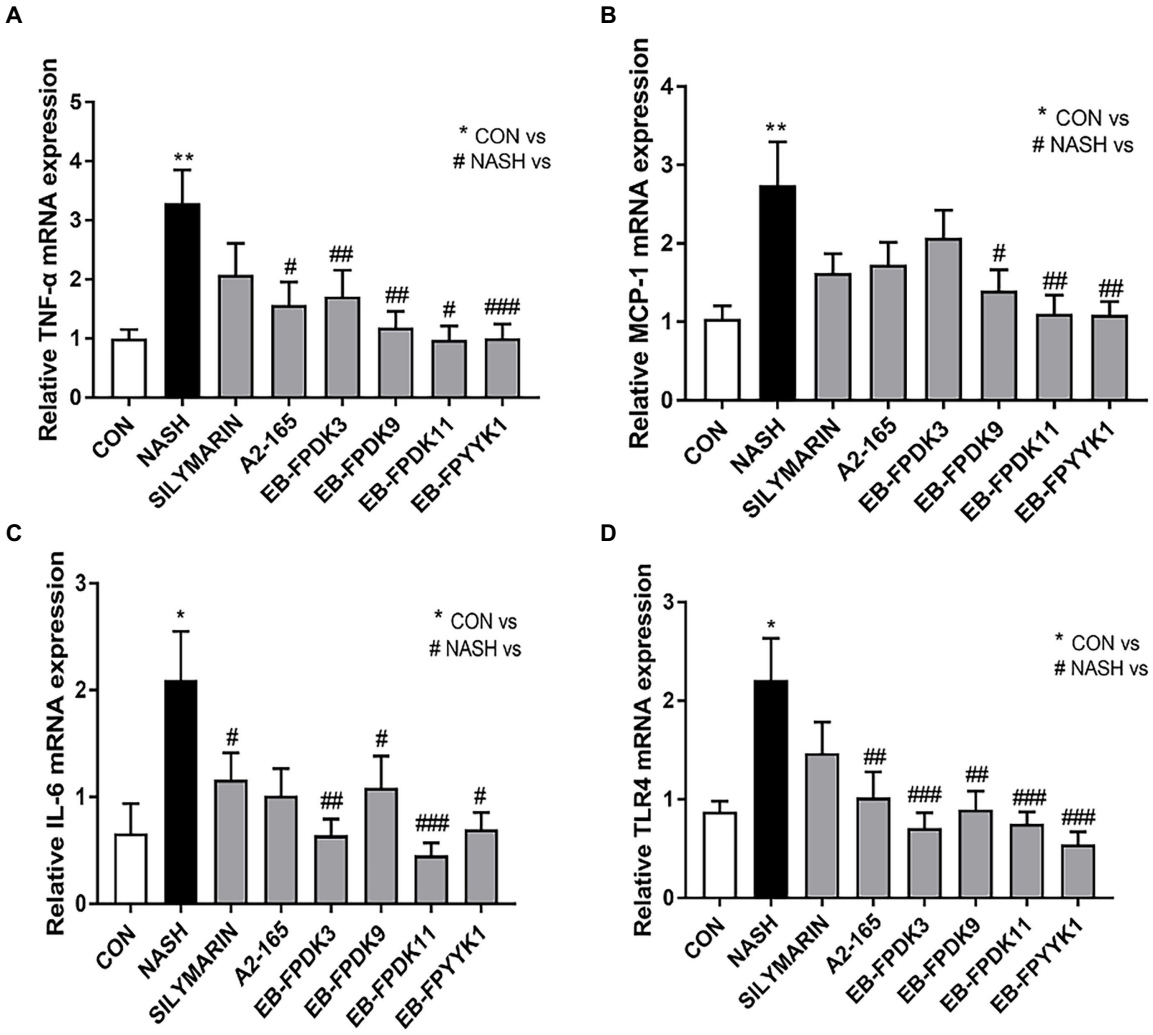
*Faeclibacterium prausnitzii* reduces the expression of inflammatory cytokines in the liver. mRNA levels of the inflammatory cytokines **(A)** TNF-α, **(B)** TLR4, **(C)** MCP-1, and **(D)** IL-6. Statistical analyzes were performed using Mann–Whitney U-test (two-tailed) **(A,B)** and Kruskal–Wallis one-way ANOVA with Dunn’s test **(C,D)**. ∗*p* < 0.05, ∗∗*p* < 0.01 versus the CON group; #*p* < 0.05, ##*p* < 0.01, ###*p* < 0.001 versus the NASH group. TNF-α, tumor necrosis factor-α; TLR4, Toll-like receptor 4; MCP-1, monocyte chemoattractant protein-1; IL-6, interleukin-6; CON, control; NASH, nonalcoholic steatohepatitis.

We also investigated the expression levels of mRNA that encode key inflammatory cytokines, namely, tumor necrosis factor-α, monocyte chemoattractant protein-1, and interleukin-6. The levels of these cytokines in the NASH group were significantly higher than those in the CON group (Figures 8A–C). However, oral administration of *F. prausnitzii* strains attenuated the severity of the hepatic inflammatory state by preventing an increase in inflammatory gene expression levels induced by NASH (Figures 8A–C). In particular, *F. prausnitzii* EB-FPDK9, EB-FPDK11, and EB-FPYYK1 showed a significant reduction in gene expression for all three cytokines compared with the NASH group. The mRNA expression of Toll-like receptor 4 was also significantly higher in the NASH group than in the CON group. However, the oral administration of *F. prausnitzii* significantly reduced the mRNA expression of Toll-like receptor 4 as induced by NASH (Figure 8D). This receptor is responsible for pathogen detection and the initiation of cytokine production. Our results, therefore, show that *F. prausnitzii* alleviated liver inflammation by regulating the expression of related genes in NASH-induced mice.

## 4. Discussion

This study establishes that healthy individuals and patients with NASH display distinct gut microbial structures. *F. prausnitzii* was among the microorganisms that were significantly lower in patients with NASH compared to healthy individuals, and we, therefore, selected this bacterium to isolate as a next-generation probiotic candidate. Indeed, treatment with *F. prausnitzii* strains in a NASH mouse model relieved NASH symptoms such as glucose homeostasis, hepatic lipid accumulation, liver damage, and liver fibrosis. Additionally, treatment with *F. prausnitzii* strains restored NASH-induced gut inflammation and altered gut permeability.

The current focus of treatment for patients with NAFLD is on dietary and lifestyle modifications, and no approved pharmacological therapies or surgical procedures exist to combat symptoms. Probiotics, prebiotics, and fecal microbiota transplants targeting the gut-liver axis are emerging as new strategies in precision medicine for both alcoholic and non-alcoholic fatty liver diseases (Bluemel et al., 2016). Probiotics are widely applied in the management of various diseases involving host-gut microbial interactions (Kim et al., 2019), and the development of effective probiotics is critical for treating liver disease (Meroni et al., 2019; Xu et al., 2021). Li et al. (2003) first reported the use of probiotics in a NASH animal model. They found that VSL#3 improved the liver histology, hepatic lipid accumulation, and serum ALT levels of mice. Since then, various studies have investigated the mechanisms underlying the effects of VSL#3 supplementation in NAFLD models (Loguercio et al., 2005; Esposito et al., 2009; Velayudham et al., 2009). Ahn et al. (2019) reported that 12 weeks of treatment with a multi-species probiotic mixture (*Lactobacillus acidophilus*, *Lactobacillus rhamnosus*, *Lactobacillus paracasei*, *Pediococcus pentosaceus*, *Bifidobacterium lactis*, and *Bifidobacterium breve*) significantly reduced the intrahepatic fat fraction and body weight of patients with obesity and NAFLD. Administration of *B. longum* reduced hepatic fat accumulation irrespective of gut permeability restoration in a NAFLD rat model (Xu et al., 2012). However, a single-strain or probiotic treatment targeting NASH, an extremely advanced form of NAFLD, is still lacking.

We selected *F. prausnitzii* as a next-generation probiotic candidate through an analysis of the gut microbiota in NASH patients, which indicated that the abundance of *F. prausnitzii* was significantly lower in patients with NASH than in healthy controls. This finding is consistent with previous reports (Da Silva et al., 2018; Iino et al., 2019; Zhong et al., 2021; Hu et al., 2022b) and suggests that *F. prausnitzii* may serve a potential role in the treatment of NASH. This approach increases the efficiency of probiotic development by targeting only the relevant microorganisms affected by NASH. Lim E. Y. et al. (2021) similarly developed an effective probiotic for alleviating menopausal symptoms by selecting probiotic candidates based on the gut microbiota dysbiosis. In this study, we found that *F. prausnitzii* strains had anti-NASH effects in a mouse model where NASH was induced *via* an HFHF diet. Treatment with *F. prausnitzii* may offer a useful therapeutic option for human patients with NASH. However, there is still a lack of information regarding the mechanisms underlying the NASH improvements observed.

In our study, the *F. prausnitzii* treatment group displayed an improvement in hepatic lipid accumulation compared to the NASH group. There is a direct relationship between liver inflammation in NASH and TG stored in the liver (Kawano and Cohen, 2013; Simental-Mendia et al., 2016). TG has been used as a marker for screening simple steatosis and NASH (Kawano and Cohen, 2013; Simental-Mendia et al., 2016). Excess ingested cholesterol is removed from the body through hepatic excretion, resulting in the liver exhibiting a lower cholesterol concentration than other tissues. The exact contribution of cholesterol consumption to NASH has not yet been determined, but it has shown a relation to NASH risk and severity (Puri et al., 2007). In addition, the induction of NASH results in a dysregulation of the hepatic cholesterol homeostasis (Ioannou, 2016).

Circulating free fatty acids are a major source of hepatic lipids in NASH models (Bradbury, 2006). The mechanisms promoting liver injury are not fully understood, but the involvement of substrates derived from adipose tissues, such as free fatty acids, leptin, and adiponectin, have been suggested (McPherson et al., 2015). Free fatty acid uptake by hepatic fatty acid transporters such as CD36 and FATP5 promotes hepatic steatosis by increasing PPAR-γ (Inoue et al., 2005). SREBP also increases the liver steatosis (Moslehi and Hamidi-Zad, 2018); its activation induces the expression of FAS and LPL, which regulate the fatty acid metabolism (Kim and Spiegelman, 1996). In this study, we found that *F. prausnitzii* strains regulated the expression of genes related to hepatic steatosis in NASH mice. This suggests that the anti-NASH mechanism of *F. prausnitzii* may inhibit lipid accumulation by regulating the genetic source of hepatic steatosis.

Disruption of the gut barrier results in a leaky gut that allows harmful substances to pass through mucosal tissues and leads to several diseases, including inflammatory bowel disease, celiac disease, and type 1 diabetes (Groschwitz and Hogan, 2009). Gut permeability is increased in patients with NAFLD patients compared to healthy controls and is associated with hepatic steatosis (De Munck et al., 2020). NASH is characterized by varying degrees of steatosis and aggressive inflammation (Hansen et al., 2017). The inflammatory response is a critical component leading to subsequent liver damage (Budick-Harmelin et al., 2008; Henao-Mejia et al., 2012). Changes in gut microbiota composition can alter gut barrier function, complementing the progress and advancement of liver disease (Plaza-Díaz et al., 2020). Our further analysis of cecum microbiota composition revealed that the intestinal microbial community was distinct between the normal and NASH groups, and EB-FPDK11 exhibited a distinct difference between the NASH and EB-FPDK9 group (Supplementary Figure S3A). In the alpha-diversity analysis, both Shannon index and Chao1 index were significantly lower in the NASH group than in the normal group. However, no significant difference was observed in the group treated with the two strains (Supplementary Figure S3B–C). The linear discriminant analysis (LDA) effect size algorithm (LEfSe) identified 11 biomarkers at the genus level in the gut microbiome of EB-FPDK9-treated group, such as *Parabacteroides* and *Odoribacter* (Supplementary Figure S3D), which have been reported to be positively correlated with the mRNA expression of tight junction proteins such as ZO1 and occludin (Koh et al., 2020; Zhao et al., 2020; Jiang et al., 2023). The abundance of *Lachnospiraceae_NK4A136_group* was significantly higher in the EB-FPDK11-treated group than in the NASH group (Supplementary Figure S3E), and this genus has been reported to play a role in maintaining the integrity of the intestinal barrier (Ma et al., 2020). In addition to *Lachnospiraceae_NK4A136_group*, *Gemella*, the proportion of which was increased in the EB-FPDK11-treated group, has reported to be positively correlated with the expression of tight junction proteins (Hu et al., 2023). The altered microbiota in the *F. prausnitzii*-treated group may have influenced the strength of the gut barrier, indicating a potential connection between the two. Several studies have reported that *F. prausnitzii* shows a strong anti-inflammatory activity (Sokol et al., 2008; Miquel et al., 2015; Lopez-Siles et al., 2017). This study recorded an improvement in inflammation and gut barrier function in the *F. prausnitzii* treatment groups when compared to conditions in the NASH group. Considering these results in combination, we can speculate that *F. prausnitzii* may improve liver histology *via* the restoration of gut barrier function that alleviates liver inflammation. However, the mechanisms underlying such gut barrier function modulation remain unexplored and deserve further research.

Another possible scenario is that *F. prausnitzii* can produce short-chain fatty acids (SCFAs) by breaking down undigested dietary fibers (Zhou et al., 2021). SCFAs include acetate, propionate, and butyrate, and their ability to alleviate the symptoms of hepatic diseases has been demonstrated *in vivo* and *in vitro*. In a mouse model of NAFLD, butyrate supplementation *via* gut microbiota attenuated hepatic steatosis *via* AMPK (adenosine 5′-monophosphate-activated protein kinase)-dependent SREBP-1c transcriptional inactivation; this effect reduced the expression of lipogenesis-related genes such as FAS and SCD1 (Zhao et al., 2021). In addition, Deng et al. (2020) reported that acetate similarly reduced liver steatosis and inflammation *via* AMPK activation in a mouse model of NASH and *in vitro* (Deng et al., 2020). Therefore, we expected the SCFA levels to decrease in the NASH model and increase in the strain-treated group. However, contrary to our expectations, in the two groups inoculated with the strain, it was observed that the levels of the three SCFAs in the cecum increased in the NASH group compared with those in the normal group, and then recovered to levels similar to those in the normal group (Supplementary Figure S4). Generally, studies in the field of liver diseases suggest that SCFAs are metabolically beneficial (Mattace Raso et al., 2013; Jin et al., 2015) and protect against gut inflammation (Kles and Chang, 2006). On the contrary, Rau et al. (2018) reported that patients with NASH had higher levels of SCFAs and more number of SCFA-producing bacteria in their fecal samples than the control group. This study established an association between increased levels of SCFAs and the progression of disease, along with immune characteristics. In patients with NASH, the increased levels of SCFAs has been linked to a decrease in the count of resting regulatory T cells (CD4 + CD45RA + CD25+) and an increase in the ratio of T helper 17 cells to resting regulatory T cells in peripheral blood (Rau et al., 2018). The study suggests that the higher prevalence of SCFA-producing bacteria in the feces of patients with NAFLD could contribute to disease progression by sustaining low-grade inflammatory processes that influence immune cells in circulation, thereby affecting peripheral target organs such as the liver or gut barrier (Rau et al., 2018). They also hinder the activity of adenosine monophosphate-activated protein kinase, leading to the buildup of hepatic free fatty acids. Similarly, the increased levels of SCFAs observed in NASH model in our study may have contributed to the persistence of an inflammatory state affecting the liver or gut barrier. The administration of *F. prausnitzii* may have played a role in restoring these processes to a normal state. The findings obtained in our study were from the caecum and thus may have limitations in terms of evaluating systemic physiological phenomena. Nevertheless, we opine that our findings are sufficient to support the application of the strains studied in clinical trials in humans.

*F. prausnitzii* possesses exceptional anti-inflammatory properties, some of which can be attributed to the generation of the MAM protein (Quévrain et al., 2016). Past phylogenetic investigations have revealed diverse phylogroups of *F. prausnitzii* strains (Fitzgerald et al., 2018; Hu et al., 2022a). Interestingly, signature MAMs derived from different F. prausnitzii phylogroups exhibit varied anti-inflammatory properties (Auger et al., 2022). These findings suggest that the MAM protein can be used as a distinctive marker for characterizing *F. prausnitzii* strains as probiotics. The MAM proteins identified from the four strains showed genetic variations in their sequences (Supplementary Figure S5A). Homology modeling of the MAM proteins showed structural differences among *F. prausnitzii* strains (Supplementary Figure S5B). Although we could not shed light on all the differences in the probiotic efficacy of each *F. prausnitzii* strain from this study, MAM protein may explain the differences in the efficacy of *F. prausnitzii* as probiotics. Moreover, Xu et al. demonstrated that MAM proteins derived from *F. prausnitzii* can restore the structure and function of the intestinal barrier by regulating the tight junction pathway and expression of ZO-1 (Xu et al., 2020). MAMs have the ability to suppress Th1 and Th17 immune responses and NF-kB activation (Sokol et al., 2008; Breyner et al., 2017). The reduction in serum LPS levels that result from the anti-inflammatory effects of MAMs (Xu et al., 2020) could also enhance gut barrier integrity and potentially decrease the likelihood of hepatic injury in NASH, given that inflammation is a contributing factor to the disease. Further research is required to examine the therapeutic effects of signature MAMs derived from tested strains in NASH.

In summary, our results demonstrated that *F. prausnitzii* treatment significantly ameliorated the symptoms associated with NASH in a mouse model, restoring gut barrier function. Furthermore, we investigated the potential mechanisms underlying the observed effects of *F. prausnitzii* treatment on lipid metabolism and inflammation. Despite the significance of our results, there were some limitations to our study. The findings of this study indicate the potential of *F. prausnitzii* as a next-generation probiotic agent for NASH prevention.

## Data availability statement

The data presented in this study are deposited in the NCBI Sequence Read Archive (SRA) repository (https://www.ncbi.nlm.nih.gov), accession numbers PRJNA901628 and PRJNA938166.

## Ethics statement

The studies involving human participants were reviewed and approved by the institutional review board of Hanyang University College of Medicine (IRB number: 2014–03–008-005). The patients/participants provided their written informed consent to participate in this study. The animal study was reviewed and approved by the Institutional Animal Care and Use Committee (IACUC) at Dongguk University (approval number: IACUC-2019-041-1).

## Author contributions

J-GS, DWJ, and Y-DN conceived and designed the project. DL, S-YJ, HRB, M-GH, S-NL, and H-JK provided an experimental supplement and technical support and managed the animal study. J-HS and YL conducted data analysis. J-HS, YL, and E-JS wrote the manuscript. All authors read and approved the final version of the manuscript.

## Funding

This work was supported by the Main Research Program (grant number E0170600-07) of the Korea Food Research Institute, funded by the Korean Ministry of Science and Information & Communication Technology.

## Conflict of interest

Authors YL, DL, S-YJ, HRB, M-GH, S-NL, and J-GS were employed by Enterobiome Inc.

The remaining authors declare that the research was conducted in the absence of any commercial or financial relationships that could be construed as a potential conflict of interest.

## Publisher’s note

All claims expressed in this article are solely those of the authors and do not necessarily represent those of their affiliated organizations, or those of the publisher, the editors and the reviewers. Any product that may be evaluated in this article, or claim that may be made by its manufacturer, is not guaranteed or endorsed by the publisher.

## Footnotes

^1^ https://dataview.ncbi.nlm.nih.gov/object/PRJNA901628?reviewer=1lnsfagkg65tcoc2833c8usaff
