# Supplementary material for: Faecalibacterium prausnitzii prevents hepatic damage in a mouse model of NASH induced by a high-fructose high-fat diet
Source: Front Microbiol. 2023 Mar 16;14:1123547. doi: 10.3389/fmicb.2023.1123547 (PMC10060964; doi:10.3389/fmicb.2023.1123547)
Supplement: Supplementary file 1 [file Data_Sheet_1.docx]

Supplementary Material

Faecalibacterium prausnitzii prevents hepatic damage in a mouse model of NASH induced by a high-fructose high-fat diet

Ji-Hee Shin^1#^, Yoonmi Lee^2#^, Eun-Ji Song^1^, Dokyung Lee^2^, Seo-Yul Jang^2^, Hye Rim Byeon^2^, Moon-Gi Hong^2^, Sang-Nam Lee^2^, Hyun-Jin Kim^3^, Jae-Gu Seo^2*^, Dae Won Jun^4*^, Young-Do Nam^1*^

*** Correspondence:**

Jae-Gu Seo, Ph.D.
jgseo@enteobiome.com

Dae Won Jun, M.D.
noshin@hanyang.ac.kr

Young-Do Nam, Ph.D.
youngdo98@kfri.re.kr

# Supplementary Figures and Tables

## Supplementary Figures


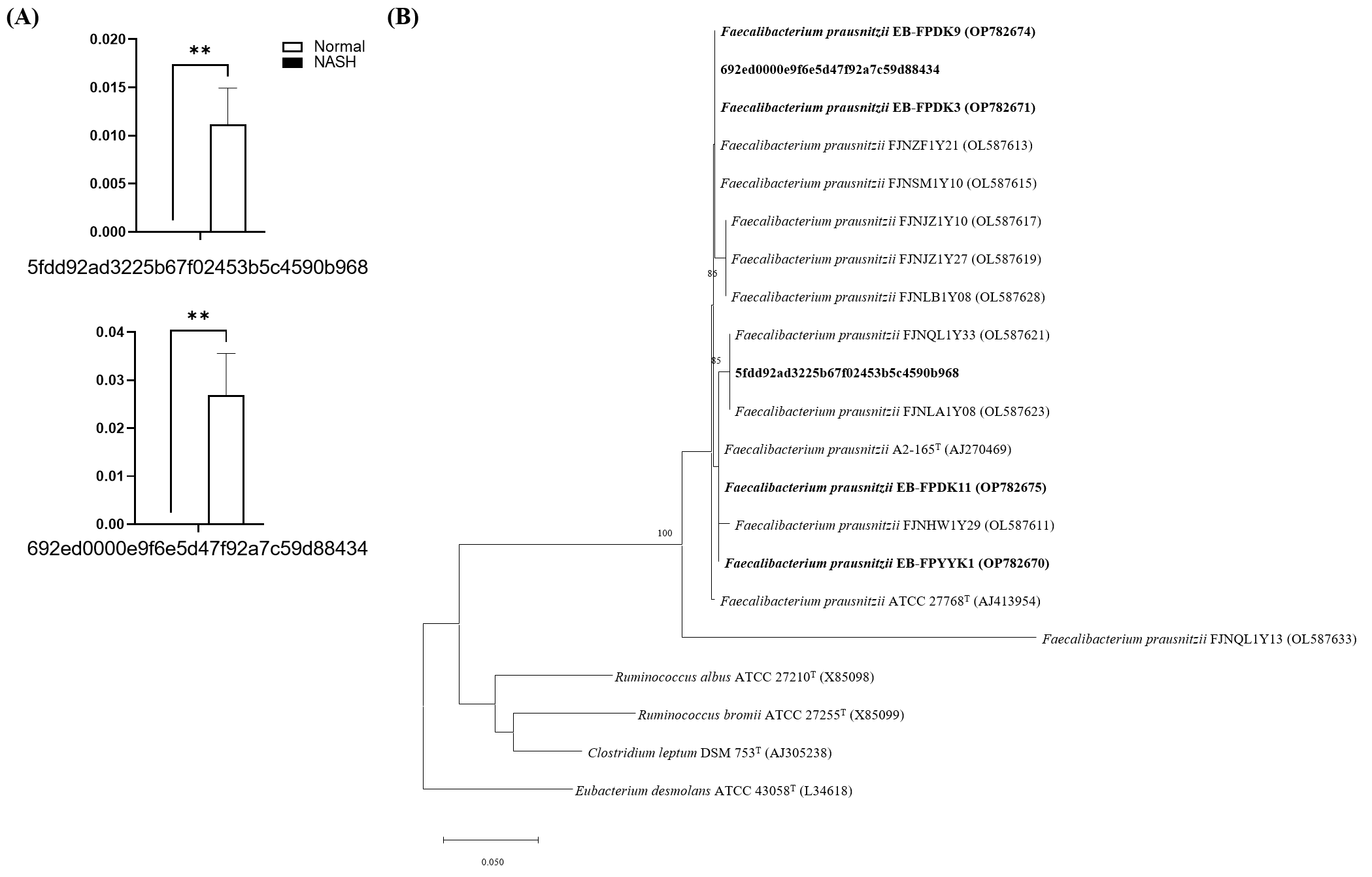


**Supplementary Figure 1.** **(A)** Two amplicon sequence variants (ASVs) in *Faecalibacterium prausnitzii* displaying significant differences in relative abundance between groups (Mann–Whitney U test; ** p ≤ 0.01). **(B)** Neighbor-joining phylogenetic tree showing the relationship between *F. prausnitzii* strains and the two ASVs in *F. prausnitzii* specifically identified in healthy individuals compared with those in patients with NASH. The tree was constructed using MEGA-X software package with the neighbor-joining method for distance analysis (Jukes-Cantor algorithm). Bootstrap values above 80% (expressed as a percentage of 1,000 replications) are shown at branching points. *Ruminococcaceae* members were included as a reference, and *Eubacterium desmolans* ATCC 43058T was used to root the tree. The two ASVs and *F. prausnitzii* strains in this study are highlighted in bold typeface. Sequence accession numbers are shown in parentheses. The scale bar indicates the number of substitutions per site.

**
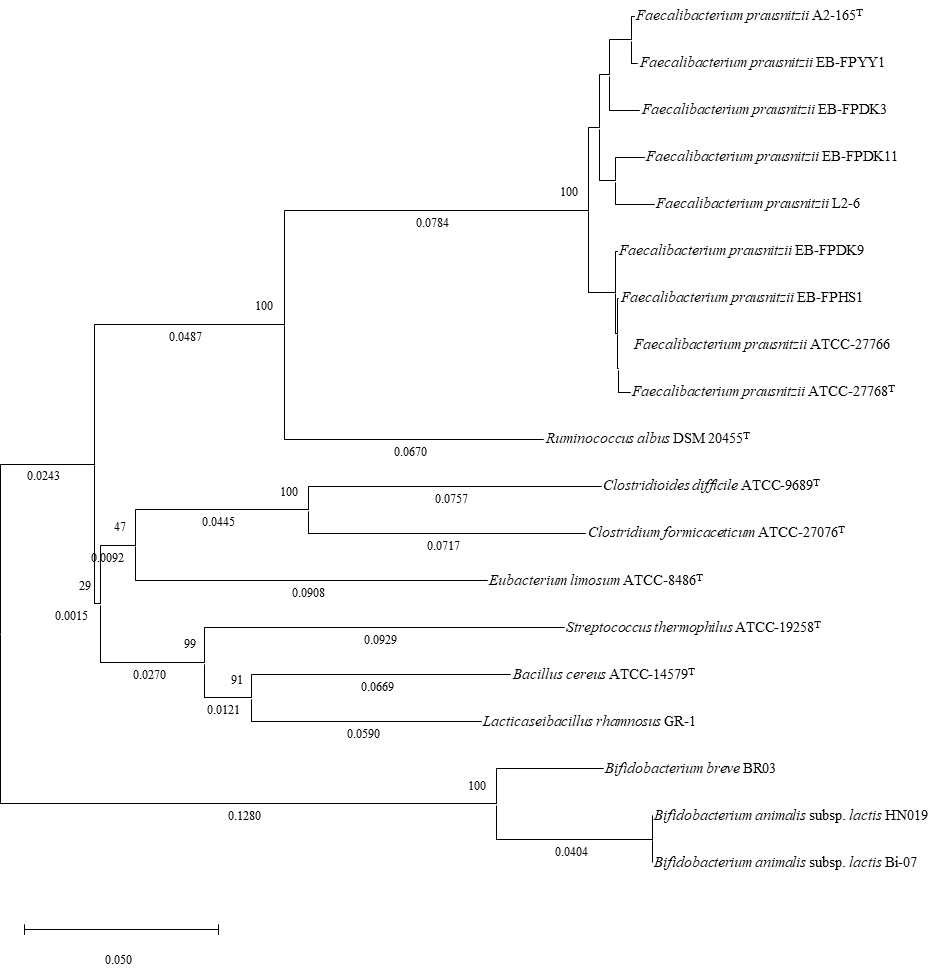
**

**Supplementary Figure 2.** Phylogenetic tree of four *F. prausnitzii* strains. The phylogenetic analysis was performed with MEGA-X, and a phylogenetic tree based on the 16S rRNA gene was constructed using the neighbor-joining method with 1000 bootstraps. Our analysis revealed that the four strains clustered with species of the genus *Faecalibacterium*. The evolutionary distances (scale bar) were computed using the Kimura 2-parameter method and are in the units of the number of base substitutions per site.


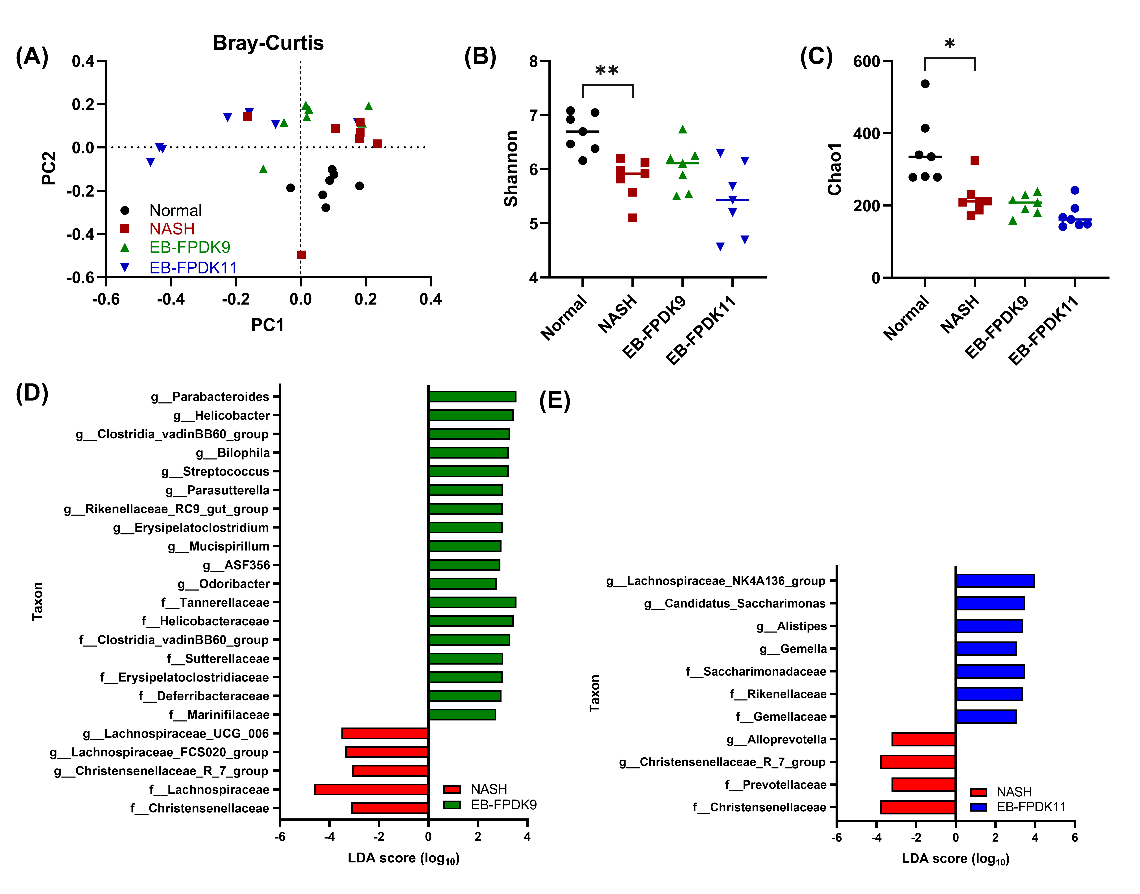


**Supplementary Figure 3. (A)** Beta- and **(B-C)** alpha-diversity of cecal microbiota in normal, NASH-induced mice, and mice treated with EB-FPDK9 and EB-FPDK11. **(D-E)** LDA score plot from the LEfSE analysis of the cecal microbiota in NASH-induced mice and mice treated with EB-FPDK9 and EB-FPDK11. Microbial taxa shown were significant (p < 0.05) and had an LDA score of >2, which was considered to indicate a significant effect size.


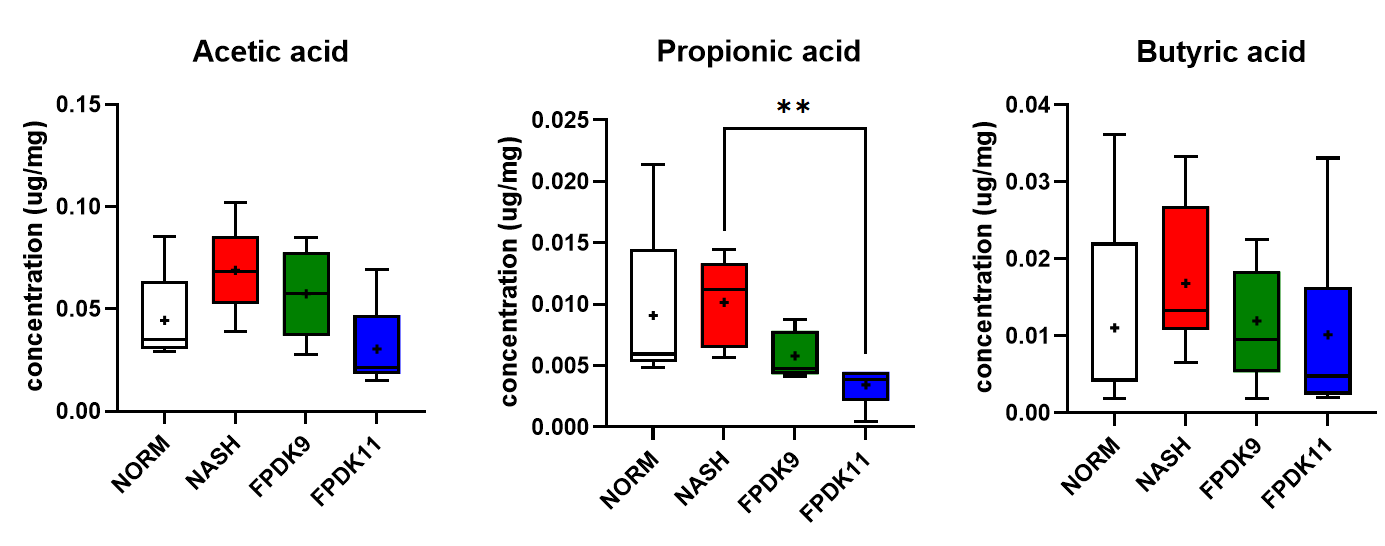


**Supplementary Figure 4.** Levels of acetic acid, propionic acid, and butyric acid in cecum. **p<0.01.

**
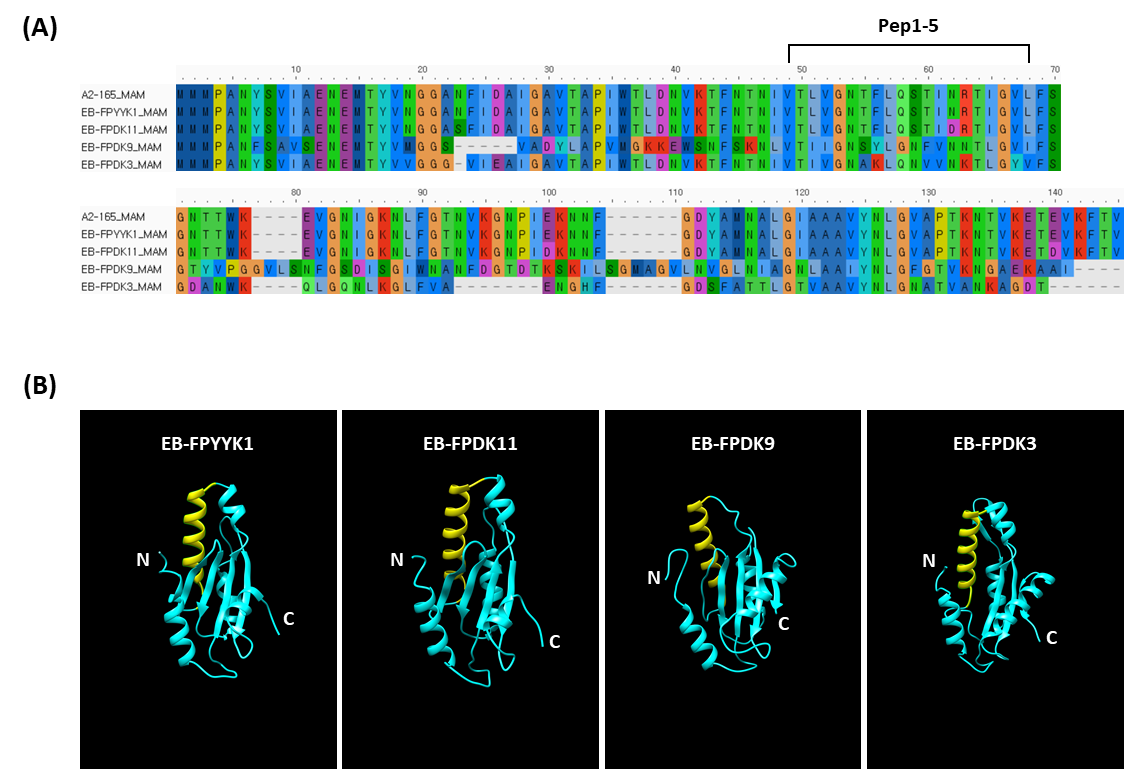
**

**Supplementary Figure 5.** Sequence alignments of MAM proteins derived from *F. prausnitzii* strains **(A)**. Three-dimensional homology models of MAM proteins by Modeller program **(B)**. The region Pep1-5 (49-68) is marked in yellow.

## Supplementary Tables

Supplementary Table 1. Antibiotic susceptibility of the *Faecalibacterium prausnitzii* type strain and isolates collected from healthy human feces

| Anti-biotics | Minimum inhibitory concentration (MIC, mg/L) | | | | | |
| --- | --- | --- | --- | --- | --- | --- |
|  | CLSI breakpoint for anaerobes | A2-165 | FPDK3 | FPDK9 | FPDK11 | FPYYK1 |
| PTZ | ≥ 128/4 | > 256/4 (R) | 32/4 (S) | 32/4 (S) | > 256/4 (R) | > 256/4 (R) |
| CTZ | ≥ 128 | 64 (I) | 16 (S) | 128 (R) | 128 (R) | 128 (R) |
| CHL | ≥ 32 | 64 (R) | 8 (S) | 32 (R) | 8 (S) | 256 (R) |
| CLI | ≥ 8 | ≤ 0.125 (S) | ≤ 0.125 (S) | ≤ 0.125 (S) | ≤ 0.125 (S) | ≤ 0.125 (S) |
| MEM | ≥ 16 | > 64 (R) | > 64 (R) | > 64 (R) | > 64 (R) | > 64 (R) |
| MXF | ≥ 8 | 16 (R) | > 32 (R) | 32 (R) | 32 (R) | > 32 (R) |
| MTZ | ≥ 32 | 4 (S) | 1 (S) | < 0.25 (S) | 0.5 (S) | 2 (S) |
| CIP | ≥ 4 | 32 (R) | 32 (R) | > 32 (R) | 16 (R) | 32 (R) |

PTZ: piperacillin-tazobactam, CTZ: ceftizoxime (3rd gen), CHL: chloramphenicol, CLI: clindamycin, MEM: meropenem, MXF: moxifloxacin (4th gen), MTZ: metronidazole, CIP: ciprofloxacin (2nd gen).
